# Sensing of p53 and EGFR Biomarkers Using High Efficiency SERS Substrates

**DOI:** 10.3390/bios5040664

**Published:** 2015-10-28

**Authors:** Peter Owens, Nigel Phillipson, Jayakumar Perumal, Gerard M. O’Connor, Malini Olivo

**Affiliations:** 1 Centre for Microscopy and Imaging, National University Ireland, University Road, Galway, Ireland; 2 School of Physics, National University Ireland, University Road, Galway, Ireland; E-Mails: N.Phillipson2@nuigalway.ie (N.P.); gerard.oconnor@nuigalway.ie (G.M.O.); 3 Bio-Optical Imaging Group, Singapore Bioimaging Consortium, Agency for Science Technology and Research (A*STAR), 11 Biopolis Way, #02-02 Helios 138667, Singapore; E-Mails: Jayakumar_Perumal@sbic.a-star.edu.sg (J.P.); malini_olivo@sbic.a-star.edu.sg (M.O.)

**Keywords:** Raman spectroscopy, protein sensing, surface-enhanced Raman scattering, biomarker

## Abstract

In this paper we describe a method for the determination of protein concentration using Surface Enhanced Raman Resonance Scattering (SERRS) immunoassays. We use two different Raman active linkers, 4-aminothiophenol and 6-mercaptopurine, to bind to a high sensitivity SERS substrate and investigate the influence of varying concentrations of p53 and EGFR on the Raman spectra. Perturbations in the spectra are due to the influence of protein–antibody binding on Raman linker molecules and are attributed to small changes in localised mechanical stress, which are enhanced by SERRS. These influences are greatest for peaks due to the C-S functional group and the Full Width Half Maximum (FWHM) was found to be inversely proportional to protein concentration.

## 1. Introduction

The development of ultrasensitive and rapid approaches to detect protein biomarkers at very low concentrations in a physiological environment represents a challenge in nano-medicine. Currently, the most widely used method for analysis of low levels of protein in basic research and clinical diagnostics is the Enzyme-Linked Immunosorbent Assay (ELISA). ELISA detection methodologies commonly employ sensitive fluorescence based immunoassays on protein arrays but require lengthy procedures and are hindered by limited multiplexed detection [1]. In terms of diagnostic protein assays, Raman spectroscopic techniques with high sensitivity and multiplexing capabilities may offer competition to fluorescence based ELISA methods. Raman spectroscopy provides a sharp label free fingerprint specific to the molecule, enabling multiplexed detection. Using Surfaced Enhanced Raman Spectroscopy (SERS) methods can in some cases surpass the measurement sensitivity of fluorescence. Although intrinsic Raman spectra are inherently weak, the scattering cross-section can be greatly enhanced by positioning of the molecular species of interest near a nanostructured metal surface (typically gold or silver) [2,3]. By tuning the incident laser frequency to the plasma frequency of the metal assembly, leading to Surfaced Enhanced Resonance Raman spectroscopy or SERRS, an overall enhancement of up to 7–8 orders of magnitude of the Raman signal is possible [4]. Measurement sensitivities of up to 10^−13^ M have been reported and more flexible multiplexing protocols have been developed due to the very narrow spectral width of Raman peaks (typically 10–100 times narrower than fluorescence peaks) [5].

**Figure 1:**
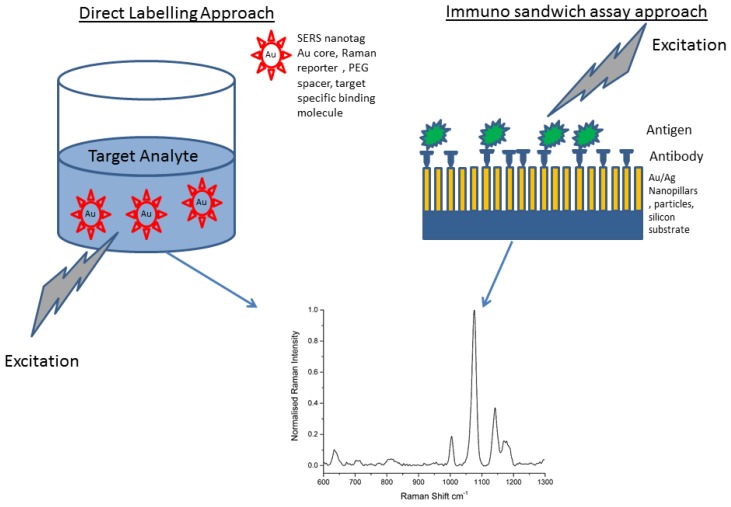
Generalised schematic showing methods for producing SERS signals from target analytes. The direct method uses metal nanoparticles bound to Raman reporters which are then bound to a specific binding molecule, *i.e.*, an antibody. These can then be used to visualise nanoparticle uptake by cells invitro for example. The second approach uses an immunolabelling strategy that uses SERS to enhance the raman signal directly from biomarker-antibody interactions.

Numerous applications of SERS in biological assays have been reported ranging from multimodal photodynamic and theranostic probes [5,6,7,8], Imaging [9], spectral analysis [10,11,12] to NIR probes capable of multiplexed detection [7,13]. Of the many methods in the literature that specifically deal with bioanalysis, two general approaches are followed. The first uses a direct labelling approach where the target analyte is mixed with a colloid of SERS optimised nanotags which then act to enhance the Raman signal of the Raman Reporter [14,15,16,17,18,19,20]. Significant developments have been made in the design and synthesis of bioconjugated nanotags, with a high degree of control on size and shape which control the plasmon frequency and Raman cross section. A number of potential bioconjugation schemes exist for SERS nanotags. One scheme involves direct immobilisation of Raman reporter molecules and antibodies onto the surface of the metal (typically gold) nanoparticles. Another approach involves conjugation of the antibody via a spacer molecule such as Polyethylene Glycol [20]. The distance to the metal nanoparticle is critical and only those labelled moieties close enough will experience significant SERS enhancement. However, reproducibility and quantification of the Raman responses from this approach can be problematic. The second approach utilises immunoassays that rely on the recognition of biomarkers with antibodies that are conjugated to SERS substrates [1,21,22,23,24]. While direct label-free approaches are more convenient than extrinsic SERS labelling, the design and synthesis of stable and well-defined metal array assemblies is still a significant challenge. In the case of trace protein analysis, low concentration high molecular weight biomolecules have limited SERS sensitivity and selectivity can be hampered by a high degree of overlap of Raman bands [25]. A comparison of these general approaches is given in Figure 1.

An alternative to direct sensing of biological analytes is to use an indirect approach. In this case a SERS based immunoassay is followed but now the concentration is measured in terms of frequency shifts in the Raman spectra that are related to mechanical perturbations [26,27]. In this approach, it is proposed that the Raman frequencies of an antibody-conjugated SERS-active molecule (or *linker*) are influenced when bound to a target antigen. The change in frequency was attributed to structural deformations which arise due to the binding event between the antibody and SERS active molecule. These structural deformations were modelled as a form of a nano mechanical stress. In this way, it was found that the concentration of the target analyte could be linked to a red shift of specific frequencies of the linker Raman spectrum. Essentially the linker bound to the substrate acts as an immobiliser for the antibody, and at the same time acts as an efficient Raman probe, the signal then being enhanced by the SERS effect.

Two target analytes, used as important biomarkers in human cancer research are p53 protein and Epidermal Growth Factor Receptor (EGFR). Two target analytes, used as important biomarkers in human cancer research are p53 protein and Epidermal Growth Factor Receptor (EGFR). One of the most widely studied DNA-binding proteins, in relation to cancer, is the p53 protein, mutations of which are found in more than 50% of all tumours. The p53 protein is at the centre of the cellular network that protects organisms against the spread of tumours, most of which are related to alteration of p53 expression. Therefore p53 is regarded as a valuable prognostic marker whose detection at high sensitivity may considerably contribute to early diagnosis of cancers [28,29]. A number of cancers are characterised by over-expression of (EGFR), a membrane protein which mediates cell growth, proliferation and differentiation in multiple tissues. Mutations involving EGFR lead to its constant activation, which produces uncontrolled cell division. EGFR exists on the cell surface and is activated by binding to its specific ligands such as Epidermal Growth Factor (EGF). EGF can be found in macrophages, urine, saliva, milk, and plasma [9,30,31].

In this work, we aim to develop robust methods for the determination of p53 and EGFR concentration using high sensitivity SERS substrates in resonance conditions [9,28,29,30,31].

## 2. Experimental Section

4-Aminothiophenol (4-ATP), 2-Naphthalene thiol (2NT), phosphate buffered saline, NHS (N-hydroxy succinimide, CAS 6066-82-6), EDC (N-(3-Dimethylaminopropyl)-N′-ethylcarbodiimide hydrochloride, CAS 25952-53-8), Glycine (CAS 56-40-6), Ethanol (spec grade CAS 64-17-5) and 6-MP (6 Mercaptopurine), were obtained from Sigma Aldrich, Ireland and used as received. Ag SERS substrates were obtained from Danish company Silmeco [32]. These substrates comprise of a layer of circa 600 nm high nanopillars on a silicon base, the localised surface plasmon resonance peak being 780 nm. The active substrate area was 25 mm^2^. Substrates were obtained in batches of 5, packaged in a nitrogen atmosphere to prevent early oxidation. Wildtype recombinant human p53 protein in aqueous buffered solution (catalog number 556439, 53 kDa) and Purified mouse anti human p53 antibody (554294, 0.5 mg/mL) were obtained from Becton Dickinson and used according to the manufacturers guidelines. No further purification steps were performed. Active human EGFR protein fragment (catalog number ab 155726, 95 kDa) and Anti-EGFR antibody (catalog number ab 2430, 0.2 mg/mL) were obtained from Abcam and used according to the manufacturers guidelines. No further purification steps were performed.

### 2.1. Functionalisation of Substrates

**Figure 2:**
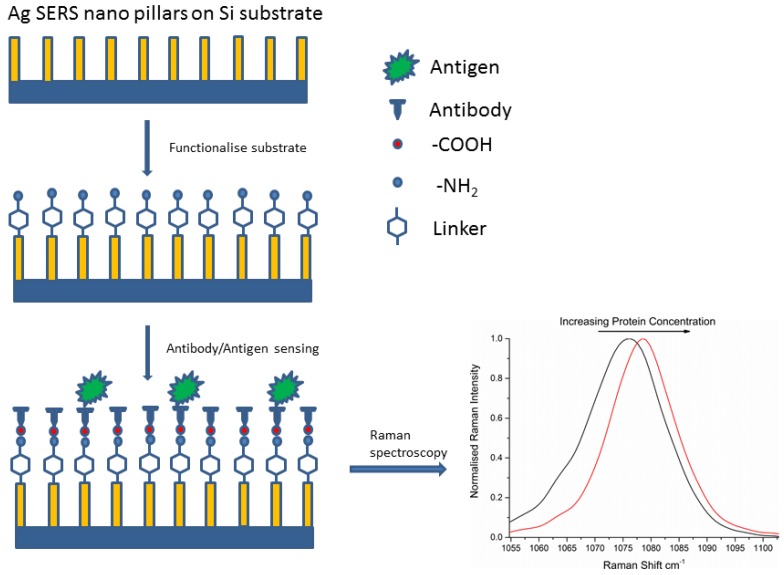
Functionalization of a Ag nanopillared substrate with linker and covalently bound antibody. With increasing amounts of target antigen, we observe a shift in the CS peak to longer wavenumbers and a corresponding decrease in the FWHM, resulting from increased mechanical stress in the system.

Substrates were first rinsed gently in 100% ethanol, as an initial cleaning step. These substrates were then incubated in either 4-ATP or 6-MP (1 mM) for one hour. The substrates were then rinsed 5 times in 100% ethanol to remove the excess/unbound linker molecules. One substrate with linker molecule only was set aside at this point. 171 mM and 427.5 mM solutions of EDC and NHS were prepared separately in 0.01 M PBS. 20 µL of Antibody (either anti-p53 or anti-EGFR) solution (0.5 mg/mL) was added to 0.5 mL of EDC and 0.5 mL of NHS. The mixture was placed under vortex for 5 min. The substrates were then covered with the EDC/NHS/Antibody mixture and allowed to incubate for 2 h. The purpose of EDC was to activate the carboxyl terminal on the surface of the antibody structure. NHS greatly improves the coupling efficiency of the reaction. After 2 h the bioconjugated substrates (antibody covalently attached to the linker-coated Ag surface) were rinsed with PBS to remove unbound antibody solution. The reaction was then blocked with EDC/NHS activated Glycine (1 mM Glycine made up in 50:50 by volume mixture of EDC/NHS) and left overnight. The substrates were then rinsed with PBS and at this point an antibody only substrate was set aside from the others. A number of concentrations of protein were prepared by serial dilution and a 20 µL of each protein sample was placed on top of the antibody conjugated substrate and left to incubate for 1 h. After 1 h the substrates were rinsed in PBS buffer to remove the excess unbound protein and Raman spectra were measured immediately. All parts of this protocol were performed at room temperature. A schematic for the substrate functionalisation is given in Figure 2. The model protein-antibody systems measured are shown in Table 1.

**Table 1:** Protein-Linker model systems studied.

Protein	Raman Active Linker
p53	ATP
EGFR	6MP
EGFR	ATP

### 2.2. Raman Microscopy

Raman microscope SERS measurements were performed in reflection mode with a Raman microscope (Witec Alpha 500) using 785 nm excitation. The detector grating used was 1200 lines/mm. Spectra were collected using an Andor Ixus cooled charge coupled device (−60 °C). A 100× objective lens (NA 0.9) delivered the laser beam and collected the back-scattered light. Rayleigh scattering was blocked with the appropriate notch filter. The laser spot diameter was ~1 µm with a laser power of 0.05 mW (785 nm) used. The Instrument calibration was verified using the maximum signal from a silicon standard at 520 cm^−1^ and spectral resolution was approximately 0.8 cm^−1^. Typical measurements were performed with a 1 s integration time and for each substrate spectra were taken at multiple positions (25) across the substrate. We expect that the substrate surface contains a mixture of free linker and protein-bound linker molecules. By recording a collection of spectra in the form of a large area scan and averaging these spectra, we obtained an average of the response from free linker molecules and protein bound linker molecules. Cosmic ray reduction (when applicable), background corrections, spectrum averaging and Savitzky-Golay smoothing (4 point, 3rd order) were carried out using Project 4 software Witec. Normalisation and graphical analysis was performed using Origin version 9.1. All spectra described are plotted relative to the excitation wavelength.

## 3. Results and Discussion

**Figure 3:**
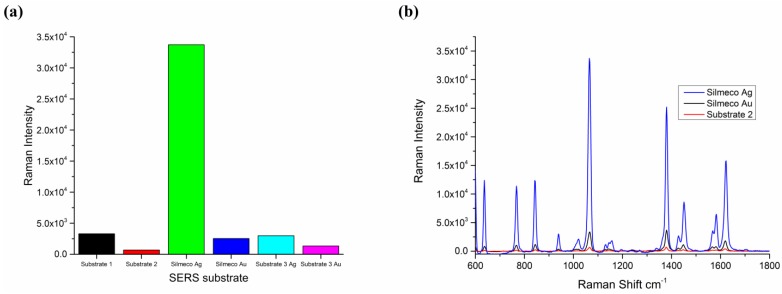
(**a**) Comparison between Silmeco Ag substrates, and a range of other commercial substrates performed at this laboratory. Substrate 1: nanoparticle; Substrate 2: nanopillar; Substrate 3: laser fabricated with either Ag or Au. Data based on maximum intensity obtained from 1064 cm^−1^ peak using 785 nm excitation. Laser power used was 0.06 mW; (**b**) SERS spectrum of 2NT after incubation on a range of substrates, showing prominent peak at 1064 relative cm^−1^, which was used for all substrate comparisons. The Silmeco Ag substrate shows a very large increase Raman intensity due to plasmon resonance effects at 785 nm excitation and nanopillar leaning enhancements.

Silmeco silver pillar SERS substrates tested using 10^−6^ M 2-Naphthalene-thiol (2NT) were found to yield a massive improvement in Raman signal intensities and superior reproducibility over a number of competitor and in-house developed substrates. Figure 3 shows the comparison in Raman intensity for number of commercial substrates (including the Silmeco Au), recorded under strict control of measurement conditions. Based on the very large improvement in signal intensity, silver Silmeco substrates were chosen for development of the SERRS based immunoassay for p53 and EGFR. The reason for the very large increase in SERS signal intensity is due to the nanopillar design of the substrate and also that due to the surface tension between pillars during liquid evaporation, the nanopillars lean toward each other, forming clusters and inducing near-field coupling effects or “hotspots” [33]. Also substrates fabricated from silver gave a further Raman intensity enhancement using 785 nm as this excitation wavelength was closest to the local surface plasmon resonance peak [34].

**Figure 4:**
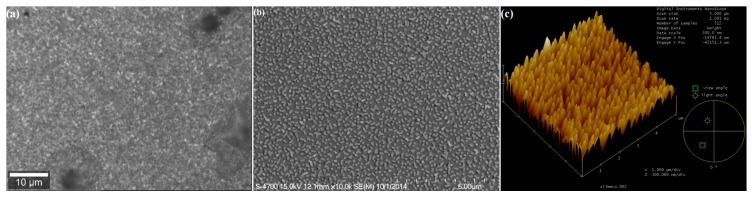
(**a**) Ag functionalised substrate, p53 protein on antibody-ATP-Ag surface. Reflected light image captured using 100× air objective lens. Bright spots show layer of Ag nanoparticles on the surface of the substrate; (**b**) Top view SEM image showing nanopillars on substrate surface, scale bar on bottom indicates 5 micron; (**c**) AFM image of Silmeco substrate showing surface topography.

Figure 4 shows a range of views of a typical substrate. The bright spots in the reflected light image relate to the Ag pillars, or groupings of pillar. In Figure 4a, the surface of the substrate is bound with protein, antibody and linker. The SEM image shows clearly the surface topography of the nanopillars pre binding (Figure 4b). Correspondingly the AFM image shows the side topographies (Figure 4c).

**Figure 5:**
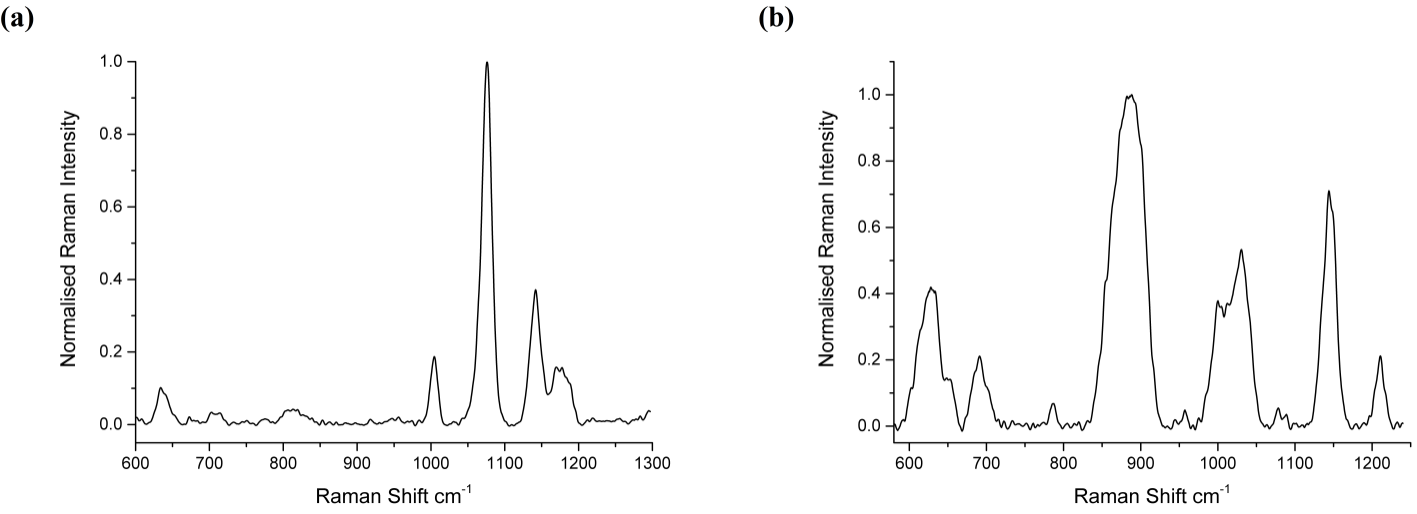
(**a**) 4-ATP Raman spectrum, on p53 bound substrate; (**b**) Raman spectrum of 6 Mercaptopurine (6MP) on EGFR bound substrate. For both spectra, 785 nm excitation and 25 point average was used.

**Figure 6:**
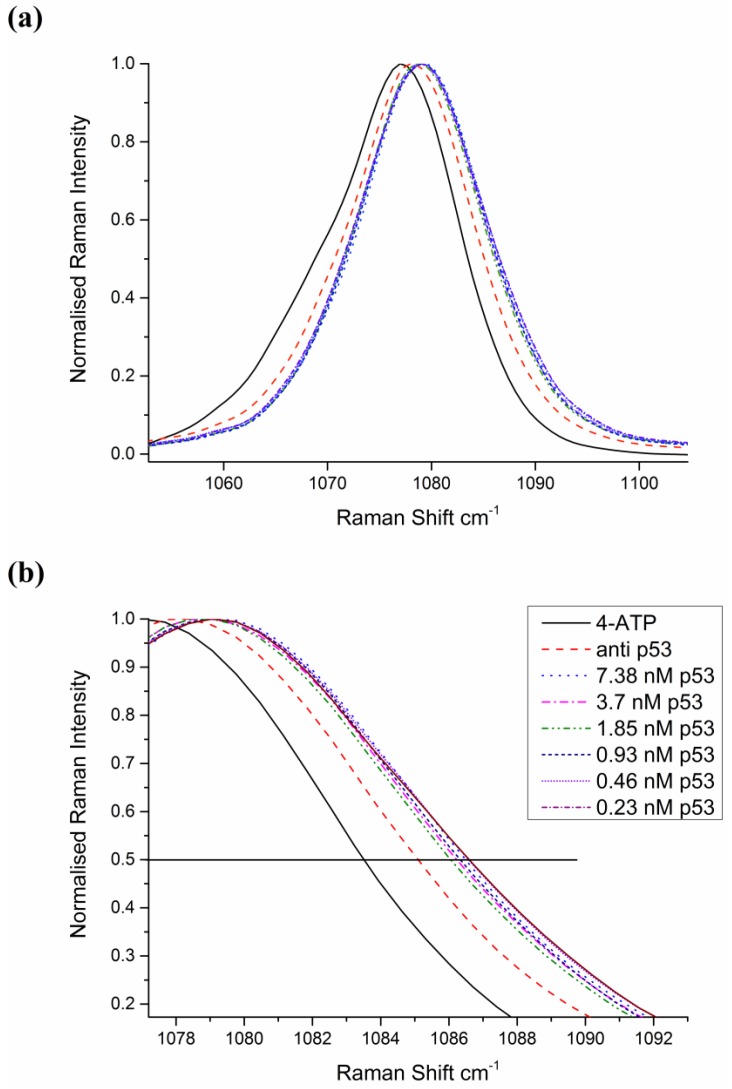
(**a**) Close up of the 1080 cm^−1^ peak for a range of concentrations of p53 protein. The lowest concentration shows the greatest red shift, but peak shift is not linear and is difficult to ascertain; (**b**) Zoom of region of forward edge of the 1080 cm^−1^ peak, showing influence of protein concentration on the spectrum of 4-ATP.

The Raman spectrum of 4-ATP from a 25 point acquisition of an area in Figure 4 is shown Figure 5a. Here the spectrum is normalised to prominent 1080 cm^−1^ peak, corresponding to the CS stretching mode, which has been assigned previously [35]. This peak was found to be particularly responsive to antibody-antigen binding and exhibits the greatest shift according to p53 concentration. Similarly, for 6MP spectra, the prominent thiol peak at 860 cm^−1^ [36,37] was found to be the most sensitive to influences from protein–antibody binding and hence all spectra were normalised to this peak. Interfering species and non-specific binding have been found to mask the SERS signal from the analyte of interest. In this work, a monolayer of linker molecules is self-assembled onto the Ag surface to prevent non-specific adsorption of molecules and improve Raman efficiencies. Furthermore, by utilising large area scans, statistical reliability and reproducibility of frequency changes are improved by accounting for spot to spot intensity fluctuations typically occurring for low abundance biomolecules. If one looks at the 1080 cm^−1^ peak for ATP in detail, as in Figure 6a, it can be seen that there is a prominent red shift due first to the ATP, and second to binding of the antibody with further shifts due to the influence of p53-anti p53 binding.

We can qualitatively observe changes in the forward edge of the 1080 cm^−1^ peak down to the lowest concentration recorded at 0.25 nM. When one expands the region of interest as in Figure 6b, it can be seen that there is a change in the forward edge of the 1080 cm^−1^ peak. Control data is given in Figure S1, showing that the method is selective to p53 protein to antibody binding.

Correspondingly, one can examine the influence of EGFR on the 860 cm^−1^ peak in the 6MP spectra, as shown in Figure S2.

**Figure 7:**
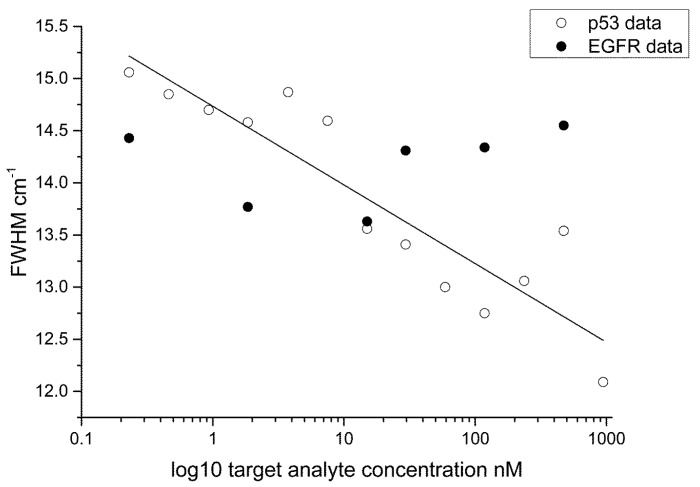
Semi-log plot of the FWHM data for p53/4-ATP. Overlaid on this plot is data for the EGFR-ATP system.

**Figure 8:**
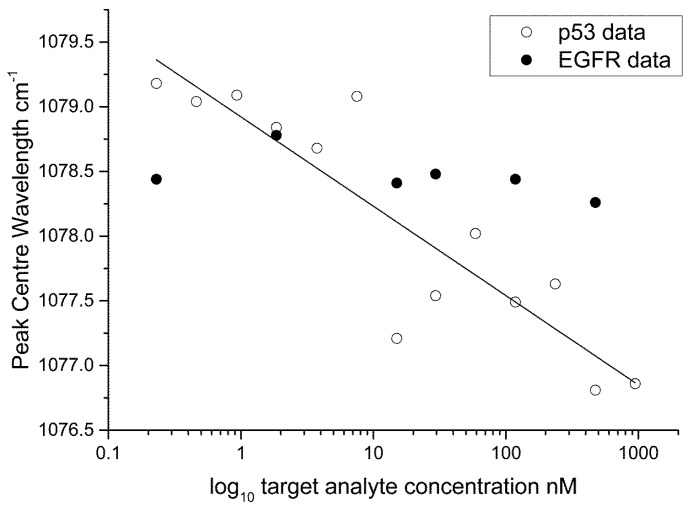
Semi-log plot showing the peak centre data for p53/4-ATP. Overlaid on this plot is data for the EGFR-ATP protein antibody system.

The FWHM (Figure 7) and peak centre (Figure 8) data are plotted as a semi-log plot (due to the large variation in concentration range) for all protein concentrations showing an exponential decrease over the range measured. A linear regression line is overlaid to show the trend in the data. We suggest that the binding between the antigen and the antibody induces structural changes in the protein moiety that in turn induce mechanical influences on the 4-ATP linker, in particular, the C–S bonding as given by the 1080 cm^−1^ peak in the 4-ATP spectrum. Peak broadening in Raman spectra has been previously linked to samples under stress. This effect has been studied in the materials sciences, in particular relating to silicon, graphene and carbon where induced mechanical stress and strain effects have been linked to frequency shifts in the Raman peaks of interest and a change in the FWHM [38,39,40]. In our case, and different to observations for Silicon, *etc.*, we find that a broadening of the 1080 cm^−1^ peak occurs at lower protein concentrations, indicating that strain in the system is reduced as the protein concentration is lowered. We believe that such changes in strain are due to changes in steric arrangement of the protein-antibody system, and are shown by broadening of the spectra with decreasing protein concentration. Additionally, the peak centre is red shifted with decreasing protein concentration further indicating reduced mechanical stress in the system. If we overlay the data for ATP-EGFR protein we see that the assay is specific to p53; a similar response for FWHM and peak centre for EGFR-ATP is not obtained. Protein interactions and their resulting structural conformations are very complicated processes. Both p53 and EGFR are comprised of electron rich aromatic lateral chains, but p53 is smaller structurally at 53 kDa mol weight compared to 97 kDa for EGFR. This size difference coupled to the difference between the amino and diazonium binding sites for 6MP and 4-ATP respectively influences the efficiency of the linker to antibody to protein binding on the substrate. Based on our data, we believe that the interactions of linker/antibody/protein systems are very specific and only certain combinations work that result in changes in the SERS spectra. The lowest concentration used in this work compares with previous detection limits reported in previous studies using stress based nano sensors [26,27].

**Figure 9:**
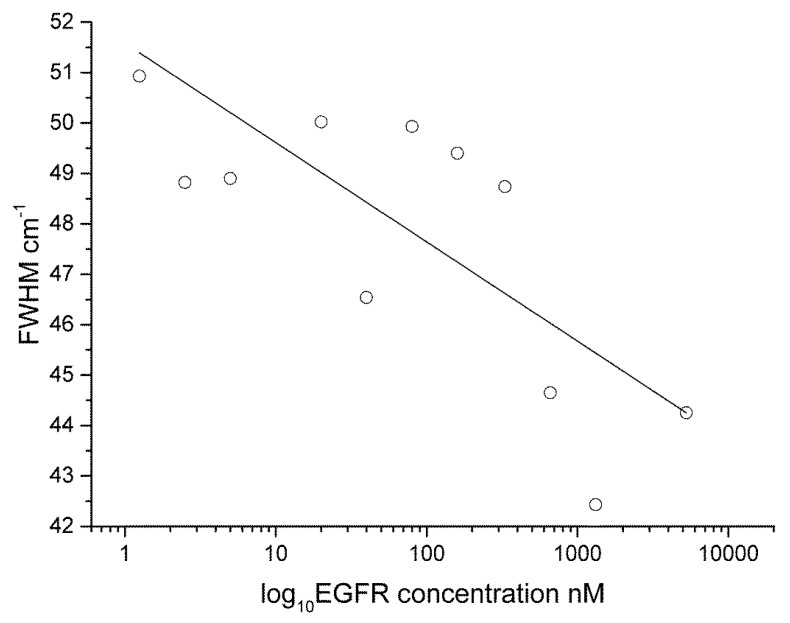
Semi-log plot showing the FWHM data from the 860 cm^−1^ peak for the EGFR/6MP system.

In the 6MP-EGFR system, we see that the system stress resulting from protein–antibody binding also results in a broadening of the FWHM (Figure 9) and follows a similar trend. Although weaker Raman scattering is observed in the 6MP-EGFR system (Figures S2 and S3), resulting in a reduction of the signal to noise ratio, we can still observe changes of FWHM. It would not be possible to detect influences of low protein concentration without the SERRS enhancement.

**Figure 10:**
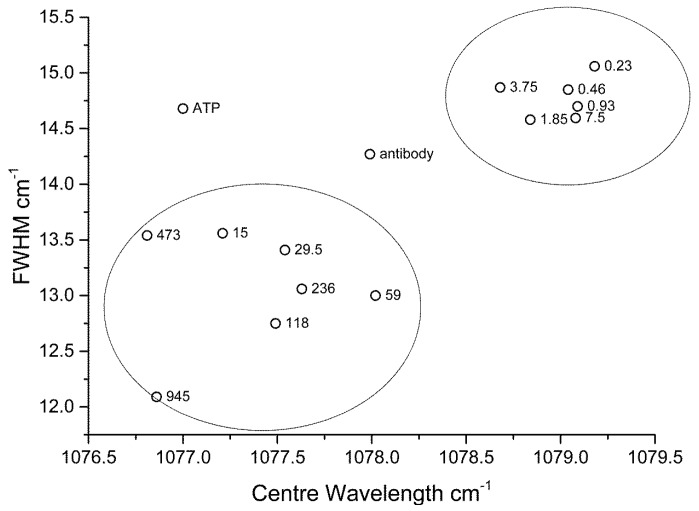
Correlation between peak centre and FWHM. Labels on the data points show the p53 concentrations (nM). The data for ATP and anti-p53 only are clear outliers.

As further validation of the stress sensor model approach, we can correlate peak centre and FWHM, which as shown in Figure 10. In this plot, clustering of low concentration data is found at longer peak centres and greater peak widths. The converse is true for samples at higher concentration, with the presence of outliers, such as that for 4-ATP and the antibody (anti-p53) only assay. By correlating the two key indicators, peak centre and FWHM as determined by our SERRS based method; we can give a direct evaluation of levels of protein in the target analyte.

## 4. Conclusions

We have shown that the influence of protein on the properties of specific peaks in Raman spectra can be measured and used as an evaluation of protein concentration. In particular the measurement of FWHM could be correlated with protein concentration in both data sets tested. We propose that measurements of changes to peak FWHM and centre are immune from system based intensity variations, particularly in the low concentration range for biosensing applications where the intensity based signal to noise is typically low. Utilising high sensitivity SERRS substrates is the key approach in this method, enabling detailed measurements at low concentration ranges. We have found that different protein/linker combinations display changes in mechanical-stress responses and this property can be exploited to enable multiplexed detection. This stress based biosensor could be used for any bio fluid that has a cancer biomarker such as oral, head and neck cancers, gastric cancers, bladder cancers, *etc*. In future work, we plan to investigate other linker protein assays and their combinations, assess the limits of detection and compare to ELISA based methods and finally develop a system for multiplexed detection. By combining sensitive detection of SERRS with the rapid readout capability of Raman based sensing, this method may be applied to the development of a point of care clinical biosensors.

## Supplementary Files

Supplementary File 1
