# Supplementary material for: Sensing of p53 and EGFR Biomarkers Using High Efficiency SERS Substrates
Source: Biosensors (Basel). 2015 Oct 28;5(4):664–77. doi: 10.3390/bios5040664 (PMC4697139; doi:10.3390/bios5040664)
Supplement: Supplementary File 1 [file biosensors-05-00664-s001.pdf]

OPEN ACCESS

biosensors

ISSN 2079-6374

www.mdpi.com/journal/biosensors/

Supplementary Information

Sensing of p53 and EGFR biomarkers using highly sensitive SERS substrates. Supplementary Information. *Biosensors*, 2015, *5*, 664-677

Peter Owens [1,†,*], Nigel Phillipson [2,†], Jayakumar Perumal [3], Gerard M. O'Connor [2] and Malini Olivo [3]

1. Centre for Microscopy and Imaging, National University Ireland, University Road, Galway, Ireland
2. School of Physics, National University Ireland, University Road, Galway, Ireland; E-Mails: N.Phillipson2@nuigalway.ie (N.P.); gerard.oconnor@nuigalway.ie (G.M.O.)
3. Bio-Optical Imaging Group, Singapore Bioimaging Consortium, Agency for Science Technology and Research (A*STAR), 11 Biopolis Way, #02-02 Helios 138667, Singapore; E-Mails: Jayakumar_Perumal@sbic.a-star.edu.sg (J.P.); malini_olivo@sbic.a-star.edu.sg (M.O.)

† These authors contributed equally to this work.

* Author to whom correspondence should be addressed; E-Mail: peter.owens@nuigalway.ie; Tel.: +353-91-494036.

Figure S1 shows the differences in FWHM between three treatment groups as follows.

1. ATP bounds substrate with anti-P53 antibody and treated with 2 nM EGFR protein.
2. ATP bound substrate with anti-p53 antibody treated with 2 nM p53 protein.
3. ATP bound substrate with anti-EGFR antibody treated with 2 nM p53 protein.

Figure S2a shows the normalised spectra for a range of EGFR concentrations down to 1.25 nM. It can be seen that the shift of peak centre due to EGFR binding has a greater range than for p53 (from 864 cm^{-1} to 879 cm^{-1}). In Figure S1b, the zoomed in region of the forward edge of the peak at 860 cm^{-1} is displayed, showing the influence of varying protein level on the normalised spectra.

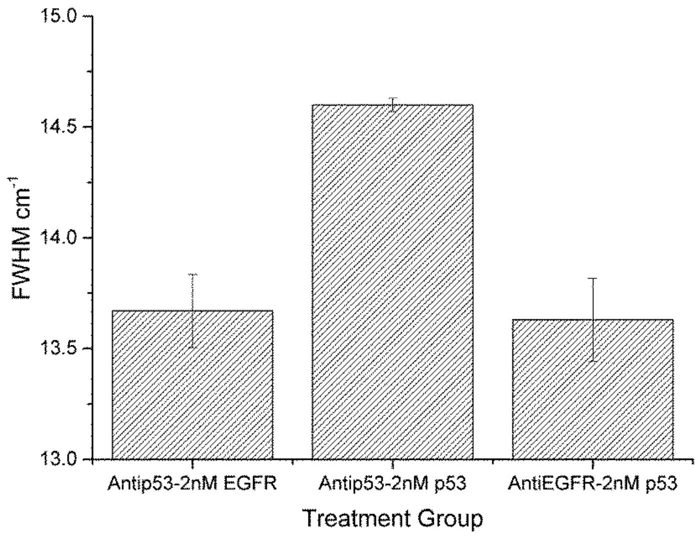

Figure S1. Control plot showing FWHM maximum data for three treatments: 1. ATP bound substrate with anti-P53 antibody and treated with 2 nM EGFR protein. 2. ATP bound substrate with anti-p53 antibody treated with 2 nM p53 protein. 3. ATP bound substrate with anti-EGFR antibody treated with 2 nM p53 protein.

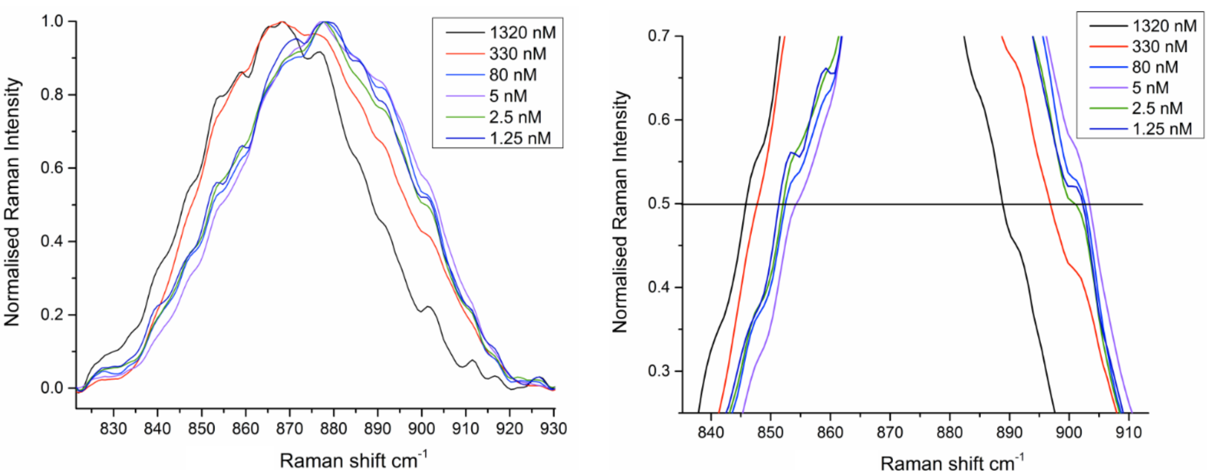

(**a**) (**b**)

Figure S2. (**a**) 6MP normalised Raman spectrum, on substrate, 785 nm excitation, 1200 lines/mm grating. The peak at 860 cm^{-1} shows the greatest shift in peak centre due to protein-antibody binding. (**b**) Zoom (subset of data removed for illustrative purposes) region of forward edge of 860 cm^{-1} peak, showing influence of EGFR concentration on the spectrum of 6MP.The black line shows the FWHM.

All 6MP-EGFR spectra were normalised to the 860 cm^{-1} peak. From this, the peak centre was calculated for each protein assay. There is a change in behaviour when the peak centre data is evaluated, as shown in Supplementary Figure S3. We now see a distinction between the higher and lower concentration levels and at the lower concentration levels, the peak centre changed slightly. The reason for this behaviour is unclear, the variation in peak centre perhaps being constrained by vibrational CS bending in 6MP.

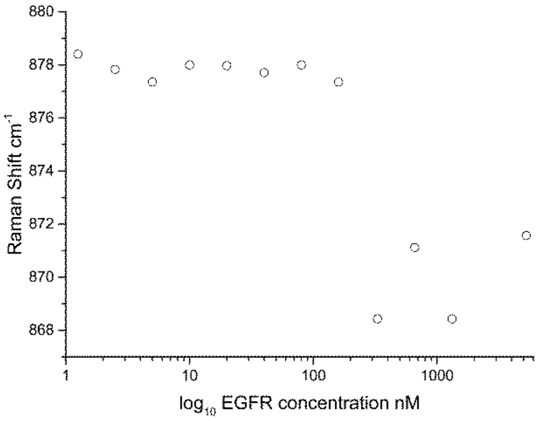

Figure S3. Semi-log plot showing the peak centre data for the EGFR/6MP system, Grating 1200, 785 nm excitation.

© 2015 by the authors; licensee MDPI, Basel, Switzerland. This article is an open access article distributed under the terms and conditions of the Creative Commons Attribution license (http://creativecommons.org/licenses/by/4.0/).
